# Supplementary material for: Case report: lipid inclusion in glomerular endothelial and mesangial cells in a patient after contrast medium injection
Source: BMC Nephrol. 2018 Mar 6;19:53. doi: 10.1186/s12882-018-0844-2 (PMC5840718; doi:10.1186/s12882-018-0844-2)
Supplement: Supplementary file 1 — Materials and Methods. (DOCX 13 kb) [file 12882_2018_844_MOESM1_ESM.docx]

Case report：lipid inclusion in glomerular endothelial and mesangial cells in a patient after contrast medium injection

Hua Su, Chen Ye, Qian Wen, Hong-Yan Zhu, Li-Xia Yi, Chun Zhang^#^

Department of Nephrology, Union Hospital, Tongji Medical College, Huazhong University of Science and Technology, Wuhan 430022, China

# Send Correspondence and Reprint Requests to:

Chun Zhang M.D., Ph.D.

Department of Nephrology,

Union Hospital,

Tongji Medical College,

Huazhong University of Science and Technology,

Wuhan 430022,

China

Email: drzhangchun@126.com

Email: [dr_suhua@hust.edu.cn](mailto:dr_suhua@hust.edu.cn) (Hua Su);

leafyechen@126.com (Chen Ye);

wenqian0524@foxmail.com (Qian Wen);

893767968@qq.com (Hong-Yan Zhu);

yilixia678119@163.com (Li-Xia Yi);

**Abstract**

**Background:** It is well-recognized that injection of iodinated radiographic contrast media (CM) sometimes causes acute renal injury via multiple mechanisms, such as vasoconstriction, toxicity on glomerular endothelium and tubular epithelium and so forth.

**Case presentation:** A 51-year-old man developed acute renal injury with proteinuria after CM administration. To our surprise, in his renal biopsy sample the myelin figure like structure was observed in glomerular endothelium and mesangial cells by transmission electron microscopy. However the patient didn’t has any clinic clues of Fabry disease and other lysosomal storage disorders. Moreover in vitro cultured glomerular endothelial and mesangial cells we found CM triggers lipid aggregation along with the increased CD36 and decreased ABCA1 abundance. Thus this patient was administrated statin to correct the aberrant lipid trafficking, 2 months later at his next visit we found his renal function partially recovered with reduced proteinuria.

**Conclusions:** Besides the well-known underlying mechanisms, CM may cause renal impairment by triggering the dysregulated transportation of lipid. Furthermore statin is suggested to be a very promising medicine to decrease side effects of CM.

**Keywords:** Case report, Contrast media, Lipidosis, Endothelial cells, Mesangial cells, CD36, ABCA1

**Background**

Contrast media (CM) administration may initiate acute renal injury and the underlying mechanisms including prolonged reduction of renal blood flow, endothelial dysfunction, dysregulation of tubular transport, etc [1]. Prophylactic intravenous hydration to avoid ischemic insult and antioxidant therapy are believed to be the major preventive strategies for contrast-induced nephropathy (CIN). In this paper, we report a CIN case morphologically presented with lipid inclusion in glomerular resident cells, especially in endothelial and mesangial cells.

**Case presentation**

A-51-year-old man was referred to urology for adrenal mass and potentially related secondary hypertension. Except for five years of uncontrolled hypertension with hypokalemia he has no other history of medication and diseases including renal disease, diabetes or other systemic disorders. His basal serum creatinine (sCr) was 1.9mg/dl with microalbuminuria, but no hematuria and cylinduria were detected at admission. Apart from the increase blood pressure there was no significant physical abnormality was identified. And his renal disorder was deem to be associated with benign nephrosclerosis caused by uncontrolled hypertension. To make a better evaluation for the adrenal mass before surgery he was subjected to enhanced adrenal CT scan with intravenous injection of iomeprol (a nonionic, monomeric iodinated contrast medium). Ahead of CM injection preventive therapies (intravenous hydration with reduced glutathione) were given to minimize the side effects of CM. The operation went extremely well and his blood pressure was well controlled after that; however his sCr was increased to 3.3mg/dl with 2+ dysmorphic hematuria and 2+ proteinuria three days later after iomeprol injection. Therefore he remained hospitalized for the elevated sCr level; unfortunately the sCr failed to drop to his basal level despite three weeks comprehensive treatment (reduced glutathione plus prostaglandin E1 administration). And the hematuria and proteinuria still persisted.

Then, he was transferred to nephrology. And he was diagnosed as acute kidney injury (AKI) superimposed on chronic kidney disease (CKD). A series of serologic and radiologic workup was ordered. Except for the elevated sCr other serologic results were unremarkable including serum potassium. Emission Computed Tomography indicated glomerular filtration rate was decreased. To get a better understanding of the nature of the renal injury the kidney biopsy was performed. On immunofluroscence microscopy no diagnostic staining of immunoglobulin and complement were detected in glomeruli, tubulointerstitium and vessels. Under light microscopy there was only mild to moderate ischemic alteration of glomeruli without proliferative lesions which is believed to be associated with the history of uncontrolled hypertension. Moreover focal mild acute tubular injury was identified presenting as the loss of brush border and isometric vacuolization of tubular epithelial cells. No coarse vacuole degeneration, the feature of hypokalemic nephrosis, was seen in tubular epithelium (data not shown). Interestingly, with the aid of transmission electron microscopy we found scattered distributed myelin figure like structure, presenting as almost-empty vacuoles circled with electron-dense membrane, in endothelium, mesangial cells and less frequently in podocyte (Fig. 1).

Combining the clinic features and pathologic findings we believed that in this case the CKD was due to moderate nephrosclerosis which could be explained by longstanding secondary hypertension. On the other hand the AKI may relate to mild acute tubular injury and scattered distributed myelin figure like structure was observed in glomerular cells.

What made us confused was the underlying etiology for the formation of myelin figure structure. As we known, zebra bodies and myelin-like figures are ordinarily seen in Fabry disease and various lysosomal storage disorders and usually infer lipid accumulation in cell bodies or subcellular organelles. However, our patient didn’t have any other systemic symptoms or family history of that genetic diseases. More importantly, his renal impairment just aggravated after CM administration. Thus we were curious to study whether CM will lead to the lipidosis in glomerular endothelial and mesangial cells. To test that, we exposed the cultured glomerular endothelial and mesangial cells to the CM, iomeprol, to evaluate whether this treatment will cause the lipid accumulation as we observed in vivo. In detail, cells were treated with iomeprol or cultured in regular medium for 1h. After that they were stained with Oil Red O or Sudan Black to detect the deposition of neutral lipids and phospholipids. Interestingly, our data revealed that iomeprol exposure promotes the lipid accumulation in the cytoplasma of endothelial and mesangial cells obviously (Fig. 2a, 3a). Next we explored the underlying mechanisms of above abnormalities. It is well-established that CD36 is an essential transporter mediating lipid uptake, whereas ATP-binding cassette transporter A1 (ABCA1) accelerates the discharge of lipid from cytosol [2,3]. Our immunofluorescence staining showed that with the treatment of iomeprol CD36 is upregulated accompanying with diminished ABCA1 intensity in both glomerular endothelial and mesangial cells (Fig. 2b, 3b) and which is the potential underlying mechanism accounting for the lipid aggregation during CM exposure.

Based on the morphologic alterations from biopsy sample and the experimental data from in vitro cultured cells, the patient was prescribed oral statins (fluvastatin, 20mg, qn) and antioxidant (Atomolam, 0.4, tid). Two months later, his next visit, we found the hematuria was resolved, and the proteinuria decreased to 1+ with improved renal function (sCr was 2.4mg/dl). (The whole timeline of this case is shown in Fig. 4)

**Conclusion**

Here we report the renal biopsy findings from an acute kidney injury case after CM injection. Conspicuously, scattered membrane-bound almost-empty vacuoles were observed in glomerular resident cells, primarily in endothelial and mesangial cells, under electron microscopy. However, this patient didn’t have any typical clinical features of lysosomal storage diseases or other hereditary disease frequently causing renal lipidosis. Also this patient didn’t take any medicine affecting the activity of lysosomal enzymes, such as amiodarone and chloroquine, which has been reported to lead to lipid accumulation in kidney [4]. Concerning the deterioration of renal function and the presence of hematuria and proteinuria were occurred just after the CM administration, we speculated glomerular lipidosis may be related to the CM in this case.

CIN is generally described as an acute worsening of renal function with an absolute increase in sCr by ≥0.3 mg/dL from baseline or a relative increase in sCr levels by ≥50% from baseline within 72 h [5]. Multiple mechanisms are involved in acute kidney injury during intravascular injection of CM. Of note, CM possesses a cytotoxic property on vascular and glomerular endothelium, as well as tubular epithelium. A few contributing factors have been suggested for these toxicities, such as the elevated formation of reactive oxygen species and oxidative stress, reduced prostaglandins, nitric oxide and adenosine production, intracellular Ca^2+^ overload and so on [6,7]. Besides its functional impairment, by scanning electron microscopy C. Gospos also observed the morphologic change of endothelium, such as, cell shrinkage, nuclear protrusion and formation of microvilli on the cell membrane [8]. Interestingly, here we observed lipid accumulation in glomerular resident cells, prominently in endothelial and mesangial cells after injection of CM. Furthermore, CM induces lipidosis in cultured glomerular endothelium and mesangial cell along with the increased CD36 and decreased ABCA1 expression. CD36, also named fatty acid translocase, is a plasma membrane transporter mediating long chain fatty acid uptake. While ABCA1, known as the cholesterol efflux regulatory protein, is a major regulator for the efflux of cellular cholesterol and phospholipid. Based on this, our findings suggest that CM induces aberrant CD36 and ABCA1 expression and which accounts for glomerular lipidosis.

Lipid overload triggers oxidation reaction, mitochondrial dysfunction, and other undesirable molecular processes which eventually results in cell and tissue damage [9]. Statin therapy showed clinical benefit in terms of preventing CIN. In a meta-analysis Ukaigwe A [10] reported that patients with type 2 diabetes, chronic kidney disease, congestive heart failure who receiving >140 ml of CM will benefit from statins therapy. Consistently, several other meta-analysis indicated that short-term, pre-procedural, potent statin (atorvastatin, rosuvastatin) therapy markedly minimized CIN risk, however the potential mechanism remains uncertain [11-13]. A few studies suggested that renoprotective effects of statin might be associated with reduction of apoptosis, oxidative stress and anti-inflammatory actions [14,15]. Currently we provide evidence that CM induces glomerular lipid aggregation along with the aberrant CD36 and ABCA1 expression. Given the fact that statin could depress CD36 and stimulate ABCA1 expression [16,17], early administration of statin might be an ideal measurement for CIN prevention by correcting the aberrant lipid trafficking. Although we do not get the direct evidence to prove CM attributes to myelin figure formation in vivo, our finding still advances the understanding of the mechanisms of CIN and renoprotective effects of statin. In the near future we will perform examinations to firmly confirm the link between CM and glomerular lipid accumulation.

**Abbreviations**

CM: Contrast Medium; CIN: Contrast Induced Nephropathy; sCr: serum Creatinine; AKI: Acute Kidney Injury; CKD: Chronic Kidney Disease; ABCA1: ATP-binding cassette transporter A1

**Declarations**

**Ethics and consent to participate**

Informed consent was obtained from each patient for renal biopsy and this study was in compliance with the Declaration of Helsinki and was approved by the local ethical committees. Otherwise, no additional ethical approval was required for this case report

**Consent to publish**

Written informed consent was obtained from the patient for publication of this case report and any accompanying images. A copy of the written consent is available for review by the editor of this journal.

**Availability of data and material**

All data generated during this study are included in this published article and its supplementary information files.

**Competing interests**

The authors declare that they have no competing interests.

**Funding**

This work was supported by grants from the National Natural Science Foundation of China (No. 81470964, No. 81570671, and No. 81522010). And this funding body supports the design of this study and collection, analysis, and interpretation of data as well as the writing and publication of this manuscript.

**Authors’ contributions**

HS, HZ and LY are responsible for the diagnostic and technical processes of the renal biopsy. HS, CY and QW participated in the organization and implementation of the in vitro studies. CZ contributed to patient management and contributed to manuscript. HS and CZ designed the study and edited and reviewed the manuscript. HS and CZ wrote the first and last drafts of the manuscript. All authors read and approved the final version of the manuscript.

**Acknowledgements**

We thanks Dr. Fan Yi from Shandong University to give us the cell line of rat glomerular endothelium as a gift.

**References**

1. Mehran R, Nikolsky E. Contrast-induced nephropathy: definition, epidemiology, and patients at risk. Kidney Int Suppl. 2006;100:S11-5.

2. Park YM. CD36, a scavenger receptor implicated in atherosclerosis. Exp Mol Med. 2014;46:e99.

3. Bielicki JK. ABCA1 agonist peptides for the treatment of disease. Curr Opin Lipidol. 2016;27:40-6.

4. Bracamonte ER, Kowalewska J, Starr J, Gitomer J, Alpers CE. Iatrogenic Phospholipidosis Mimicking Fabry Disease. Am J Kidney Dis. 2006;48:844-50.

5. Lakhal K, Ehrmann S, Chaari A, Laissy JP, Régnier B, Wolff M, Pajot O. Acute Kidney Injury Network definition of contrast-induced nephropathy in the critically ill: incidence and outcome. J Crit Care. 2011;26:593-9.

6. Heyman SN, Rosen S, Rosenberger C. Renal parenchymal hypoxia, hypoxia adaptation, and the pathogenesis of radiocontrast nephropathy. Clin J Am Soc Nephrol. 2008, 3: 288-96.

7. Sendeski MM. Pathophysiology of renal tissue damage by iodinated contrast media. Clinical and Experimental Pharmacology and Physiology. 2011;38:292-9.

8. Gospos C, Freudenberg N, Staubesand J, Mathias K, Papacharlampos X. The effect of contrast media on the aortic endothelium of rats. Radiology.1983;147:685-8.

9. Janikiewicz J, Hanzelka K, Kozinski K, Kolczynska K, Dobrzyn A. Islet β-cell failure in type 2 diabetes--Within the network of toxic lipids. Biochem Biophys Res Commun. 2015;460:491-6.

10. Ukaigwe A, Karmacharya P, Mahmood M, Pathak R, Aryal MR, Jalota L, Donato AA. Meta-analysis on efficacy of statins for prevention of contrast-induced acute kidney injury in patients undergoing coronary angiography. Am J Cardiol. 2014;114:1295-302.

11. Shehata M, Hamza M. Impact of high loading dose of atorvastatin in diabetic patients with renal dysfunction undergoing elective percutaneous coronary intervention: a randomized controlled trial. Cardiovasc Ther. 2015;33:35-41.

12. Han Y, Zhu G, Han L, Hou F, Huang W, Liu H, Gan J, Jiang T, Li X, Wang W, Ding S, Jia S, Shen W, Wang D, Sun L, Qiu J, Wang X, Li Y, Deng J, Li J, Xu K, Xu B, Mehran R, Huo Y. Short-term rosuvastatin therapy for prevention of contrast-induced acute kidney injury in patients with diabetes and chronic kidney disease. J Am Coll Cardiol. 2014;63:62-70.

13. Marenzi G, Cosentino N, Werba JP, Tedesco CC, Veglia F, Bartorelli AL. A meta-analysis of randomized controlled trials on statins for the prevention of contrast-induced acute kidney injury in patients with and without acute coronary syndromes. Int J Cardiol. 2015;183:47-53.

14. Su J, Zou W, Cai W, Chen X, Wang F, Li S, Ma W, Cao Y. Atorvastatin ameliorates contrast medium-induced renal tubular cell apoptosis in diabetic rats via suppression of Rho-kinase pathway. Eur J Pharmacol. 2014;723:15-22.

15. Wu K, Lei W, Tian J, Li H. Atorvastatin treatment attenuates renal injury in an experimental model of ischemia-reperfusion in rats. BMC Nephrol. 2014;15:14.

16. Mandosi E, Fallarino M, Gatti A, Carnovale A, Rossetti M, Lococo E, Buchetti B, Filetti S, Lenti L, Morano S. Atorvastatin downregulates monocyte CD36 expression, nuclear NF kappaB and TNF alpha levels in type 2 diabetes. J Atheroscler Thromb. 2010 30;17:539-45.

17. Shimizu T, Miura S, Tanigawa H, Kuwano T, Zhang B, Uehara Y, Saku K. Rosuvastatin activates ATP-binding cassette transporter A1-dependent efflux ex vivo and promotes reverse cholesterol transport in macrophage cells in mice fed a high-fat diet. Arterioscler Thromb Vasc Biol. 2014;34:2246-53.

**Additional files**

Additional file 1 CARE Checklist

Additional file 2 Materials and Methods

**FIGURE LEGENDS**

**Fig. 1 Myelin figure like structures are observed in glomeruli under transmission electron microscopy. a-c** Low magnification shows lipid inclusion in endothelium (arrows), mesangial cell (arrowheads) and podocyte (asterisks). (Original magnification, ×2,500) **d** Higher magnification shows almost-empty vacuoles circled with electron-dense membrane in endothelium (arrow) (Original magnification, ×5,000) **e** Higher magnification shows myelin figure like structure with empty vacuoles in mesangial cell (arrowheads). (Original magnification, ×5,000)

**Fig. 2 Lipid is accumulated in iomeprol treated glomerular endothelial cells.** Rat glomerular endothelial cells were cultured in DMEM/F12 supplemented with 10% FBS and then treated without (Ctrl) or with iomeprol (CM, 50μl/ml) for 1h. **a** Oil Red and Sudan Black staining presents lipid inclusion in endothelium in Ctrl and CM groups. **b** Immunofluorescence staining indicates CD36 and ABCA1 protein expression in Ctrl and CM groups. (Original magnification, ×400)

**Fig. 3 Lipid is deposited in glomerular mesangial cells under iomeprol stimulation.** Rat glomerular mesangial cells were cultured in Minimum Essential Medium supplemented with 10% FBS and then treated without (Ctrl) or with iomeprol (CM, 50μl/ml) for 1h. **a** Oil Red and Sudan Black staining shows lipid accumulation in mesangial cells from Ctrl and CM groups. **b** Immunofluorescence staining presents CD36 and ABCA1 protein expression in Ctrl and CM groups. (Original magnification, ×400)

**Fig. 4 The timeline of this case report.**
